# Hemorheological Parameters in Diabetic Patients: Role of Glucose Lowering Therapies

**DOI:** 10.3390/metabo11120806

**Published:** 2021-11-27

**Authors:** Katalin Biro, Gergely Feher, Judit Vekasi, Peter Kenyeres, Kalman Toth, Katalin Koltai

**Affiliations:** 1 1st Department of Medicine, Medical School, University of Pecs, Szigeti u 12, 7624 Pecs, Hungary; biro.katalin@pte.hu (K.B.); kenyeres.peter@pte.hu (P.K.); toth.kalman@pte.hu (K.T.); 2 Centre for Occupational Medicine, Medical School, University of Pecs, Szigeti u 12, 7624 Pecs, Hungary; feher.gergely@pte.hu; 3 Department of Ophthalmology, Medical School, University of Pecs, Szigeti u 12, 7624 Pecs, Hungary; vekasi.judit@pte.hu

**Keywords:** hemorheology, viscosity, red blood cell aggregation, diabetes, insulin, metformin, sulfonylureas

## Abstract

Diabetes mellitus influences several important hemorheological parameters including blood viscosity, erythrocyte aggregation and deformability. In the present study, 159 type-2 diabetic patients and 25 healthy controls were involved. Patient’s age, body weight, body mass index (BMI), smoking habits, physical activity, history of cardiovascular diseases, current antidiabetic therapy and concomitant medication were recorded. Patients were grouped according to their antidiabetic treatment with insulin, or with one or more of the following antidiabetic drugs: metformin, sulfonylureas, acarbose, or no antidiabetic therapy. Hemorheological measurements (hematocrit, erythrocyte aggregation, plasma fibrinogen, whole blood and plasma viscosity), von Willebrand factor activity, and platelet aggregation measurements were performed. Platelet aggregation was investigated with the method of Born. Plasma viscosity and red blood cell aggregation were significatly higher in diabetes. No significant difference was found in hemorheological parameters between different antidiabetic regimens. Whole blood and plasma viscosity and red blood cell aggregation correlated with glucose levels but not with HbA1C levels. In conclusion, plasma and whole blood viscosity, as well as red blood cell aggregation appear to be associated with concurrent hyperglycemia, but not with the quality of glycemic control or the applied antidiabetic treatment. Platelet aggregation induced by ADP or epinephrine does not seem to be associated with diabetes even at subthreshold doses.

## 1. Introduction

Diabetes mellitus resulting in micro-and macrovascular complications is one of the major risk factors for cardiovascular disease. Hemorheological alterations have been associated with diabetes mellitus and diabetes related conditions as hyperglycemia, hyperinsulinemia and insulin resistance [1,2,3]. Increased red blood cell aggregation in diabetic patients has been described by several studies. Poor glycemic control was found to be an important determinant of excessive erythrocyte aggregation. Our previous results showed significant elevation in red blood cell aggregability in patients with abnormal glucose tolerance in oral glucose tolerance tests during hyperglycemia [4]. Significantly higher whole blood viscosity has been found in patients with diabetes by several researchers [5]. Elevated blood viscosity plays an important role in diabetic microangiopathy by adversely affecting microcirculation [6].

Both type-1 and type-2 diabetes are characterized by diabetic thrombocytopathy. Hyperglycemia, insulin resistance or deficiency, cellular abnormalities as increased generation of thrombin and thromboxane A2, accelerated platelet turnover and associated metabolic conditions as obesity, dyslipidemia and inflammation have been identified as mechanisms of platelet dysfunction [7]. Platelet abnormalities in diabetes include reduced membrane fluidity, altered platelet shape, secretion and aggregation, increased formation of platelet-derived microparticles, increased expression of surface receptors and adhesion molecules as well as increased platelet-dependent activation of coagulation [8].

Compared to the large amount of data concerning the hemorheological consequences of diabetes, studies on the effects of antidiabetic drugs on these parameters are much less abundant.

Sulfonylureas are insulin secretagogues that stimulate insulin release from pancreatic beta cells. Early filterability studies did not show different effect on blood filterability from various kinds of sulfonylureas and insulin [9]. An inhibitory effect of certain sulfonylureas on platelet aggregation has been described by a number of authors. Gliclazide, glibenclamide, glyburide and to a lesser extent glimepiride were suggested to have platelet aggregation inhibitory properties [8,10]. On the other hand, a study by Larkins et al. reported no significant effect of gliclazide on platelet function in insulin-treated and non-insulin-treated diabetic patients [11]. A novel investigation of two population-based cohorts found decreased mean platelet volume (MPV), a cell trait partially associated with markers of platelet activity in sulfonylurea treated patients [12].

Metformin is the most prescribed drug for type 2 diabetes mellitus (T2DM) treatment. Studies investigating hemorheological properties in metformin treatment are scarce and limited in sample size. Metformin has been found to improve endothelial function [13]. Schiapaccassa et al. reported no effect of metformin treatment on blood viscosity [14]. A number of studies found that metformin affected platelet activation [15] and aggregation [16]. Metformin has been reported to decrease mean platelet volume (MPV) [12,17]. However, it was suggested that these effects were caused by improved glycemic control rather than any specific effect of metformin.

Acarbose, an α- glucosidase inhibitor is not widely used nowadays due to its relatively modest impact and the potential for significant gastrointestinal adverse effects, diarrhea and flatulence. Very limited research data is available on possible hemorheological or microcirculatory effects of acarbose therapy. In a study by Shimbakuro et al. postprandial endothelial dysfunction defined by peak forearm blood flow response and total reactive hyperemic flow was improved by a prior use of acarbose [18].

The first investigations on the hemorheological effects of insulin were performed in the 70ies and the 80ies, however, most of these studies had a limited sample size and used varying methodologies. Results concerning the effect of insulin on hemorheological parameters remained contradictory [9]. Insulin therapy reduced erythrocyte aggregation in gestational diabetes [19]. In a study by Jennings et al. the effect of intensified dietary measures and subsequent insulin therapy upon haemorheological measures was studied in Type 2 diabetic patients. Increased levels of the platelet release proteins beta-thromboglobulin and platelet factor 4 but no hemorheological changes were found [20].

## 2. Results

Diabetic patients had a significantly higher BMI than controls. They were 8 years older on average, and exercised less (Table 1). They had higher glucose and lower HDL cholesterol levels than controls. Red blood cell aggregation and von Willebrand factor activity were significantly higher in the diabetic group. Plasma viscosity was significantly higher in diabetic patients. The difference in whole blood viscosity and in fibrinogen levels between diabetic patients and healthy controls was not statistically significant. Glucose levels, but not HbA1c levels, were correlated with plasma viscosity (*p* < 0.01), whole blood viscosity (*p* < 0.05), red blood cell aggregation (M: *p* < 0.05, M1: *p* < 0.001), and von Willebrand factor activity (*p* < 0.05). In the subgroup of diabetic patients who were not treated with antiplatelet agents, while there was a trend towards higher platelet aggregability compared to controls, the difference was significant only with the lowest applied doses of collagen inducer (Table 2).

The examined hemorheological parameters of insulin-treated patients did not differ significantly from those of patients treated with oral antidiabetic drugs. Insulin-treated patients had higher blood glucose, fructosamine, HbA1c, and CRP levels compared to patients treated with oral antidiabetic drugs (Table 3).

Platelet aggregation was higher in insulin-treated patients, compared to patients treated with oral antidiabetic drugs who were not receiving any antiplatelet therapy, in cases when low doses of collagen inducer were applied (Table 4).

**Figure 1:**
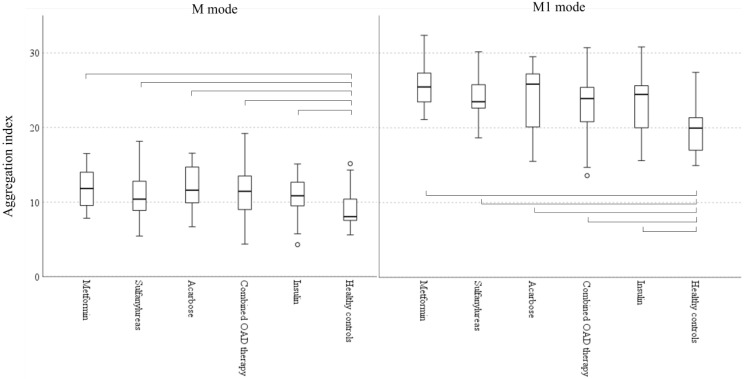
Red blood cell aggregation in patients with different antidiabetic regimens and in healthy controls (Boxplots: median, IQR and 5–95 percentiles; circle: outliers; parentheses show significant difference among groups according to Kruskal-Wallis test, *p* < 0.05). M mode: aggregation at stasis, M1 mode: aggregation at low shear.

We found no significant differences in hemorheological parameters (Figure 1), von Willebrand factor activity, or lipid and glycemic parameters between patients treated with different antidiabetic therapy (Table 5).

## 3. Discussion

Similar to results commonly found in earlier studies, we found higher plasma viscosity and red blood cell aggregation in diabetic patients compared to healthy controls [5,21]. Whole blood viscosity is mainly determined by hematocrit, plasma viscosity, erythrocyte aggregation and red blood cell deformability [5], which was reflected in correlations between these parameters in the present study. On the other hand, we did not find a clear difference in whole blood viscosity between diabetic patients and healthy controls, unlike several pioneer researchers of hemorheology in earlier decades [5]. This might be associated with basic changes in the therapy of diabetes and concomitant vascular diseases when compared to the 70ies, such as the use of statins and the much wider use of antiplatelet agents. Fibrinogen is an acute phase reactant and independent cardiovascular risk factor. Although fibrinogen has been found to be elevated in type-2 diabetic patients in several studies [22,23,24,25,26], in our present study we did not find a significant difference in fibrinogen levels between diabetic patients and controls, despite the presence of several other aggravating factors in the diabetic group, such as higher age, a higher percentage of vascular diseases, and higher body weight and BMI. Concomitant lipid-lowering therapies may influence fibrinogen levels in diabetic patients, as both statin and fibrate monotherapies have been found to decrease fibrinogen levels [27].

De Silva et al. found that patients treated with sulfonylureas had higher fibrinogen concentration than patients who were treated with insulin, biguanides, or sulfonylurea plus biguanides [28]. Contrary to these results, no significant difference in fibrinogen levels was found between different antidiabetic regimens in the present study.

High platelet reactivity in diabetes has been described in several studies. Platelets in diabetic patients were reported to undergo rapid consumption due to hyperreactivity even to subthreshold stimuli [29]. While chronic hyperglycemia has been associated with in vivo platelet activation and platelet hyperreactivity, tight metabolic control has been found to reduce urinary thromboxane metabolites. Increased levels of von Willebrand factor in the circulatory system seem to correlate with an increase in platelet activation in diabetes [30]. In our study, we applied such subthreshold doses of inducers, however, a significant difference in platelet aggregation between diabetic and healthy people was seen only with the lowest applied dose of collagen. In a recent study performed with light aggregometry, partially similar to our results, platelet aggregation induced by ADP, collagen, or epinephrine did not appear to be consistently associated with diabetes [12].

## 4. Materials and Methods

### 4.1. Patients and Methods

A total of 159 type-2 diabetic patients (83 females, 76 males, mean age: 60 ± 10 years) and 25 healthy controls (15 females, 10 males, mean age: 52 ± 12 years) were involved in this study. Patient’s age, body weight, BMI, smoking habits, physical activity, case history of cardiovascular diseases, family history of diabetes and cardiovascular diseases, current antidiabetic therapy, and concomitant medication were recorded. Physical activity/week was assessed by self-report. Hours spent engaging in exercise at least equivalent in intensity to vigorous walking were recorded. Patients were either treated with insulin or with one or more of the following antidiabetic drugs: metformin, sulfonylureas, acarbose. A total of 12 diabetic patients were involved, who were not treated with any antidiabetic drugs. Patients who were treated both with insulin and with oral antidiabetic drugs were excluded from the study.

### 4.2. Hemorheological Measurements

Hematocrit, erythrocyte aggregation, and von Willebrand factor measurements were performed at room temperature (22 ± 1 °C). Whole blood viscosity, plasma viscosity, and platelet aggregation measurements were performed at 37 °C within two hours after venipuncture. Plasma fibrinogen levels were measured by the Clauss method.

#### 4.2.1. Plasma and Whole Blood Viscosity

Venous blood was collected into lithium-heparin coated Vacutainer tubes. Plasma and whole blood viscosity were measured in a Hevimet 40 capillary viscosimeter (Hemorex Ltd., Budapest, Hungary). Samples were centrifugated at 1500× *g* for ten minutes to obtain plasma. A 1.0 mL sample was injected into the capillary tube of the viscosimeter. The flow of the fluid was detected optoelectronically. Shear rate and shear stress were calculated from the flow curve. Viscosity was calculated as a function of these parameters, according to Casson’s principle. Whole blood viscosity values, calculated at 90 s^−1^ shear rate, were used for the presentation of results.

#### 4.2.2. RBC Aggregation

Venous blood samples were collected into lithium-heparin coated Vacutainer tubes. RBC aggregation was assessed in a Myrenne aggregometer (MA-1 Aggregometer, Myrenne GmbH, Roetgen, Germany), according to the light transmission method of Schmid-Schonbein et al. To assess M and M1 mode aggregation, 30 μL of blood was first sheared at 600 s^−1^ in order to disperse pre-existing aggregates. M mode was detected by decreasing the shear rate rapidly to zero. In the case of M1 mode, low shear rate was achieved. The aggregation indices, M and M1, were calculated from the surface area below the light-intensity curve, in a 10 s measurement period.

#### 4.2.3. Hematocrit

Venous blood was collected into lithium-heparin coated Vacutainer tubes. Samples were centrifugated in hematocrit capillaries at 12,000 rpm for five minutes, in a microhematocrit centrifuge (Hemofuge, Heraeus Instr., Hanau, Germany).

### 4.3. Measurement of von Willebrand Factor

Von Willebrand factor (vWf) activity was assessed by quantitative direct-enzyme immunoassay (Shield Diagnostics Ltd., Dundee, UK). The wells of microtiter strips were coated with a preparation of a purified monoclonal antibody that binds to a functional epitope of the vWf antigen. Plasma was added, and vWf bound to the plates. After further incubation and washing, a third layer was introduced that was comprised of a horseradish-peroxidase-labelled mouse anti-human monoclonal anti-vWf conjugate to vWf. After a washing step, a substrate solution was added to trace the specifically bound antibody. A stop solution terminated the reaction. The amount of conjugate bound was measured in absorbance units. A dose-response curve was prepared from a calibrator set, according to the 4th International Standard. Activity of vWf was estimated by interpolation.

### 4.4. Measurement of Platelet Aggregability

Blood samples were collected into sodium-citrate coated Vacutainer tubes between 8 a.m. and 10 a.m., after an overnight fast. Platelet-rich plasma (PRP) was obtained by centrifugation at 150× *g* for 10 min. PRP was carefully removed. Platelet-poor plasma (PPP) was prepared by further centrifugation of the remaining samples at 2500× *g* for 10 min. Platelet aggregation was measured according to Born’s method in a 4-channel optical aggregometer (Carat TX-4, Carat Diagnostics Ltd., Budapest, Hungary), at 37 °C, within two hours after vein puncture. Platelet aggregation was evaluated considering the maximal percentage of platelet aggregation in response to 0.5, 1, 2.5, 5, and 10 µM ADP; 0.2, 0.5, 1, and 2 µg/mL collagen; and 1, 2.5, 5, and 10 µM epinephrine inducers. Spontaneous aggregation was also assessed. In the diabetic group, platelet aggregability was investigated only in a subgroup of those patients who were not under antiplatelet therapy (55 patients, 24 males, 31 females). None of the control patients were on antiplatelet therapy.

### 4.5. Statistical Analysis

The Kolmogorov–Smirnov test was used to investigate normal distribution of parameters. As the results showed mostly non-normal distribution, nonparametric tests were used. Variables were presented as median (interquartile range) or mean ± SD. The Mann–Whitney U test was used to detect differences between diabetic patients and controls, as well as patients taking oral antidiabetic drugs or insulin. The Kruskall-Wallis test was used to test hemorheological and blood-chemistry parameters between different regimens of oral antidiabetic treatments. A *p* value < 0.05 was considered statistically significant. Relationships between dichotomic variables were tested with the Chi-square test. Spearman’s correlation was used between hemorheological parameters, von Willebrand factor activity, and glucose and HbA1c levels.

## Figures and Tables

**Table 1:** Hemorheological and selected other parameters in diabetic patients and non-diabetic controls. NS: not significant.

Variables	Diabetic Patients (*n* = 159)	Healthy Controls (*n* = 25)	*p*
Body weight (kg)	83 (75–95)	72 (63–92)	*p* < 0.05
BMI (kg/m^2^)	29.4 (26.1–32.9)	24.9 (21.8–29.3)	*p* < 0.001
Physical activity/week	1 (1–2)	3 (2–3.5)	*p* < 0.001
Glucose (mmol/L)	8.0 (6.7–11.3)	4.7 (4.4–4.9)	*p* < 0.001
Triglyceride (mmol/L)	1.90 (1.30–2.82)	0.94 (0.70–1.29)	*p* < 0.001
Total cholesterol (mmol/L)	4.99 (4.31–5.71)	4.91 (4.37–5.45)	NS
HDL cholesterol (mmol/L)	1.12 (0.96–1.37)	1.37 (1.17–1.69)	*p* < 0.001
Uric acid (µmol/L)	288 (234–342)	279 (242–341)	NS
CRP (mg/L)	3.55 (1.6–6.1)	1.95 (1.0–3.43)	*p* < 0.001
Hematocrit (%)	40.8 (38.7–43.3)	41.5 (38.9–44.8)	NS
Platelet count (G/L)	240 (189–280)	260 (206–325)	NS
Whole blood viscosity (mPAS)	4.74 (4.22–5.21)	4.5 (4.13–4.94)	NS
Plasma viscosity (mPAS)	1.33 (1.28–1.40)	1.28 (1.24–1.3)	*p* < 0.001
Erythrocyte aggregation index M	11.35 (9.48–13.3)	8.05 (7.15–10.73)	*p* < 0.01
Erythrocyte aggregation index M1	24.13 (21.86–26.21)	19.95 (16.95–21.73)	*p* < 0.001
Fibrinogen (g/L)	3.44 (3.03–3.94)	3.27 (2.93–3.65)	NS
vWf activity	138 (98–182)	102 (75–117)	*p* < 0.01

**Table 2:** Platelet aggregation induced with different doses of ADP, collagen and epinephrine in diabetic patients who were not under antiplatelet therapy and in non-diabetic controls. NS: not significant.

Inducer Concentrations	Diabetic Patients (*n* = 45)	Control Group (*n* = 25)	*p* (Mann-Whitney)
ADP 10 µM	79 (69–84)	79 (69–89)	NS
ADP 5 µM	78 (64–82)	76 (62–86)	NS
ADP 2.5 µM	68 (58–76)	67 (82–80)	NS
ADP 1 µM	57 (16–77)	37 (14–72)	NS
ADP 0.5 µM	12 (4–62)	11 (2–64)	NS
Collagen 2 µg/mL	75 (67–81)	78 (69–89)	NS
Collagen 1 µg/mL	68 (60–74)	70 (35–77)	NS
Collagen 0.5 µg/mL	66 (44–75)	21 (2–76)	NS
Collagen 0.2 µg/mL	58 (6–70)	2 (0–53)	*p* < 0.01
Epinephrine 10 µM	81 (68–89)	85 (64–92)	NS
Epinephrine 5 µM	71 (55–77)	71 (43–79)	NS
Epinephrine 2.5 µM	68 (39–83)	68 (13–73)	NS
Epinephrine 1 µM	65 (29–77)	60 (5–70)	NS
Spont. aggr	4 (1–19)	2 (0–4)	NS

**Table 3:** Hemorheological and other parameters in diabetic patients who were either on insulin, or on oral antidiabetic treatment. NS: not significant.

Variables	Insulin Therapy (*n* = 33)	Oral Antidiabetic Therapy (*n* = 124)	*p* (Mann-Whitney)
Age (years)	59 (50.5–67)	60.5 (54–68)	NS
Sex	61% male	52% male	NS (Chi Square Test)
Body weight (kg)	85 (78–94)	82 (73–95)	NS
BMI (kg/m^2^)	30.4 (27.2–33.1)	29.3 (25.9–32.9)	NS
Physical activity/week	1.5 (1–2.25)	1 (1–2)	NS
Glucose (mmol/L)	11.1 (7.7–13.4)	7.7 (6.5–9.6)	*p* < 0.001
HbA1c (%)	7.89 (6.65–8.74)	6.56 (5.91–7.57)	*p* = 0.001
Triglyceride (mmol/L)	1.76 (1.19–2.30)	1.90 (1.41–2.86)	NS
Total cholesterol (mmol/L)	5.11 (4.61–5.89)	4.98 (4.26–5.68)	NS
HDL cholesterol (mmol/L)	1.14 (1.03–1.54)	1.10 (0.92–1.30)	NS
Uric acid (µmol/L)	297 (240–338)	286 (234–350)	NS
CRP (mg/L)	5.0 (3.0–8.1)	3.0 (1.3–6.0)	*p* < 0.01
Hematocrit (%)	41.5 (40.0–44.2)	40.8 (38.6–43.1)	NS
Platelet (G/L)	244 (192–283)	235 (189–279)	NS
Whole blood viscosity (mPAS)	4.8 (4.36–5.39)	4.72 (4.29–5.21)	NS
Plasma viscosity (mPAS)	1.35 (1.28–1.44)	1.33 (1.28–1.39)	NS
Erythrocyte aggregation index M	10.7 (9.5–12.7)	11.5 (9.5–13.6)	NS
Erythrocyte aggregation index M1	24.7 (20.0–25.6)	24.0 (22.0–26.7)	NS
Fibrinogen (g/L)	3.58 (3.16–4.25)	3.40 (2.96–3.83)	NS
vWf activity	138 (119–200)	138 (87–178)	NS

**Table 4:** Platelet aggregation in diabetic patients who were not under antiplatelet therapy and were treated either with insulin or with oral antidiabetic medicines. NS: not significant.

Inducer Concentrations	Insulin Therapy (*n* = 15)	Oral Antidiabetic Therapy (*n* = 31)	*p* (Mann-Whitney)
ADP 10 µM	79 (70–85)	78 (68–84)	NS
ADP 5 µM	80 (72–84)	76 (63–82)	NS
ADP 2.5 µM	72 (59–76)	66 (51–76)	NS
ADP 1 µM	67 (40–77)	36 (14–70)	NS
ADP 0.5 µM	10 (6–59)	13 (4–65)	NS
Collagen 2 µg/mL	71 (66–79)	76 (68–83)	NS
Collagen 1 µg/mL	75 (66–82)	66 (52–72)	*p* < 0.05
Collagen 0.5 µg/mL	72 (59–76)	66 (31–71)	NS
Collagen 0.2 µg/mL	65 (45–78)	14 (3–65)	*p* < 0.05
Epinephrine 10 µM	76 (68–88)	84 (77–90)	NS
Epinephrine 5 µM	74 (69–78)	70 (32–76)	NS
Epinephrine 2.5 µM	83 (68–84)	66 (13–76)	NS
Epinephrine 1 µM	70 (65–80)	65 (9–76)	NS
Spont. aggr	6 (1–42)	4 (1–17)	NS

**Table 5:** Selected hemorheological, laboratory, and clinical parameters of patients treated with different oral antidiabetic regimens.

Variables	Metformin *n* = 16	Sulfonylureas *n* = 41	Acarbose *n* = 15	Combined Oral Antidiabetic Therapy *n* = 42	No Antidiabetic Therapy *n* = 12
Whole blood viscosity (mPAS)	4.75 (4.44–5.26)	4.80 (4.29–5.28)	4.56 (4.15–5.11)	4.67 (4.27–5.08)	4.48 (4.10 –5.58)
Plasma viscosity (mPAS)	1.35 (1.28–1.40)	1.34 (1.29–1.44)	1.31 (1.27–1.35)	1.34 (1.27–1.42)	1.26 (1.24–1.38)
Aggregation index M	11.8 (9.3–14.1)	10.4 (8.8–12.9)	11.6 (9.8–14.9)	11.5 (9.0–13.6)	12.9 (10.1–14.9)
Aggregation index M1	25.5 (22.9–27.7)	23.8 (22.6–26.2)	25.8 (19.2–27.3)	23.9 (20.8–25.5)	25.0 (23.0–29.7)
Plasma fibrinogen (g/L)	3.51 (2.96–3.74)	3.48 (3.05–4.06)	3.42 (3.01–3.61)	3.40 (2.89–3.85)	3.53 (3.06–3.78)
Triglyceride (mmol/L)	2.15 (1.37–3.08)	2.09 (1.24–3.01)	1.84 (1.46–2.57)	1.90 (1.43–2.87)	2.04 (1.51–3.94)
Cholesterol (mmol/L)	4.72 (4.19–5.26)	5.19 (4.42–5.72)	4.78 (4.16–5.74)	4.80 (3.96–5.62)	4.48 (4.04–6.03)
HDL cholesterol (mmol/L)	1.18 (1.06–1.31)	1.05 (0.88–1.44)	1.07 (0.98–1.45)	1.07 (0.88–1–24)	1.15 (1.02–1.52)
vWf activity	115 (35–171)	132 (118–185)	141 (78–240)	129 (88–177)	118 (36–156)
Glucose (mmol/L)	8.04 (5.6–9.3)	7.7 (6.8–10.0)	6.5 (5.9–8.2)	7.9 (6.7–11.8)	6.8 (6.4–8.3)
HbA1c (%)	6.7 (5.08–7.3)	6.5 (6.1–7.2)	6.03 (5.6–8.1)	6.6 (6.1–8.0)	7.9 (6.6–8.7)
Hematocrit (%)	40.8 (39.7–44.0)	41.7 (38.6–43.1)	40.3 (38.2–45.6)	40.2 (38.0–41.7)	42.5 (38.3–45.9)
Body weight (kg)	75 (70–92)	80 (75–94)	77 (64–92)	87 (77–105)	75 (63–94)
BMI (kg/m^2^)	27.1 (25.2–31.7)	29.4 (26.0–32.5)	27.6 (25.1–30.1)	30.5 (27.5–35.8)	27.7 (23.9–32.8)
Previous diseases (%)					
Hypertension	87	92	83	90	100
Myocardial infarction	18	22	21	12	0
Angina pectoris	43	35	35	46	27
Stroke	56	32	14	37	54
Transient ischemic attack	31	12	28	23	18
Peripheral artery disease	6	7	0	7	0
Carotid stenosis	0	2	0	0	0
Venous thromboembolism	0	2	0	0	18
Average of years since diagnosis of diabetes	11	7	2	10	5

## Data Availability

Data are available on request from the corresponding author according to local policies.
